# Supplementary material for: Closed-form feedback-free learning with forward projection
Source: Nat Commun. 2026 Feb 5;17:2414. doi: 10.1038/s41467-026-69161-1 (PMC12988171; doi:10.1038/s41467-026-69161-1)
Supplement: Supplementary file 1 — Supplementary Information [file 41467_2026_69161_MOESM1_ESM.pdf]

Supplementary Information for Closed-Form Feedback-Free Learning with Forward Projection

Robert O'Shea[1*] and Bipin Rajendran[1*]

[1*]Centre for Intelligent Information Processing Systems, Department of Engineering, King's College London, Strand, London, WC2R 2LS, London, UK.

*Corresponding author(s). E-mail(s): k1930297@kcl.ac.uk; bipin.rajendran@kcl.ac.uk;

S.1 Key Equations from Main Text

For convenience, we restate key equations from the main text that are referenced throughout this supplement.

The target potential for layer l is generated as:

$$\tilde{\mathbf{z}}_l = g_l(\mathbf{a}_{l-1}\mathbf{Q}_l) + g_l(\mathbf{y}\mathbf{U}_l) \tag{1}$$

Layer weights are computed via ridge regression:

$$\mathbf{W}_l := (\mathbf{A}_{l-1}^\top \mathbf{A}_{l-1} + \lambda \mathbf{I})^{-1}(\mathbf{A}_{l-1}^\top \tilde{\mathbf{Z}}_l) \tag{2}$$

S.2 Local Learning Methods

Supplementary Figure S1 illustrates training procedures and information flows in forward-projection, local-supervision, Forward-Forward and backpropagation algorithms.

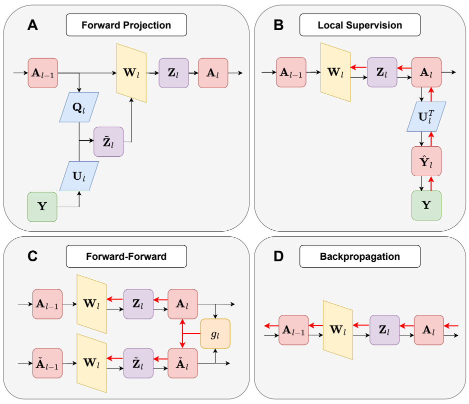

Fig. S1: Training Procedures for Forward Projection, Local Supervision, Forward Forward and Backpropagation learning algorithms for the l-th hidden layer. A: Forward Projection generates target matrix $\tilde{\mathbf{Z}}_l$ by projecting pre-synaptic inputs $\mathbf{A}_{l-1}$ by $\mathbf{Q}_l$ and labels $\mathbf{Y}$ by $\mathbf{U}_l$. Weights $\mathbf{W}_l$ are fitted by regression, generating membrane potential $\mathbf{Z}_l = \mathbf{A}_{l-1}\mathbf{W}_l$. B: In Local Supervision, an auxiliary prediction $\hat{\mathbf{Y}}_l$ is generated as a projection of the post-synaptic outputs $\mathbf{A}_l$, and $\mathbf{W}_l$ is updated by a short backward pass (red arrows). C: In Forward-Forward, "positive" and "negative" pre-synaptic activities, $\mathbf{A}_l$ and $\check{\mathbf{A}}_l$, are generated from true and spurious data-label pairs, respectively. $\mathbf{W}_l$ is updated to maximise positive activity whilst minimising negative activity. D: In backpropagation, $\mathbf{W}_l$ is updated along its gradient with respect to the backpropagated error.

S.3 Convolutional Layer Implementation for FP

We consider implementation of Forward Projection in a 2D convolutional layer over pre-synaptic input $\mathbf{a}_{l-1} \in \mathbb{R}^{1\times m_{l-1}\times R\times C}$, having m_{l-1} channels, and spatial dimensions $R\times C$ representing rows and columns. The $k_1\times k_2$ convolutional kernel positioned in row $r \in \{1,\ldots,R\}$ and column $c \in \{1,\ldots,C\}$ is represented in "flattened" form as $\mathbf{a}_{l-1,r,c} \in \mathbb{R}^{1\times(m_{l-1}k_1k_2)}$. This pre-synaptic input is projected by the constant matrix $\mathbf{Q}_l \in \mathbb{R}^{(m_{l-1}k_1k_2)\times m_l}$ to generate local target potentials $\tilde{\mathbf{z}}_{l,r,c} \in \mathbb{R}^{1\times m_l}$, such that

$$\tilde{\mathbf{z}}_{l,r,c} = g_l\left(\mathbf{a}_{l-1,r,c}\mathbf{Q}_l\right) + g_l\left(\mathbf{y}\mathbf{U}_l\right). \tag{3}$$

Weights were fitted such that

$$\mathbf{W}_l := \left(\sum_{r=1}^{R}\sum_{c=1}^{C}\mathbf{A}_{l-1,r,c}^\top\mathbf{A}_{l-1,r,c} + \lambda\mathbf{I}\right)^{-1}\left(\sum_{r=1}^{R}\sum_{c=1}^{C}\mathbf{A}_{l-1,r,c}^\top\tilde{\mathbf{Z}}_{l,r,c}\right). \tag{4}$$

Here, $\mathbf{A}_{l-1,r,c} \in \mathbb{R}^{N\times(m_{l-1}k_1k_2)}$ is a matrix representing the pre-synaptic input for the convolutional kernel in row r and column c of the l-th layer over all N training instances. Likewise, $\tilde{\mathbf{Z}}_{l,r,c} \in \mathbb{R}^{N\times m_l}$ contains target potentials for the kernel in row

r and column c of the l-th layer over all N training instances. In each task, the FP penalty parameter was fixed at $\lambda = 10$ for hidden layers and $\lambda = 1$ for output layers. To prevent numerical overflow due to large layers or training datasets, a scalar reduction factor $\tau \in (0, 1]$ may be applied to downscale $\mathbf{A}^\top\mathbf{A}$, λ, and $\mathbf{A}^\top\mathbf{Z}$ as necessary, such that:

$$\mathbf{W}_l := \left(\sum_{r=1}^{R}\sum_{c=1}^{C} \tau \mathbf{A}_{l-1,r,c}^\top \mathbf{A}_{l-1,r,c} + \tau\lambda\mathbf{I}\right)^{-1} \left(\sum_{r=1}^{R}\sum_{c=1}^{C} \tau \mathbf{A}_{l-1,r,c}^\top \tilde{\mathbf{Z}}_{l,r,c}\right). \tag{5}$$

S.4 Attention Layer Implementation for FP

We consider the implementation of Forward Projection in a transformer layer [6] over pre-synaptic input $\mathbf{a}_{l-1} \in \mathbb{R}^{R \times m_{l-1}}$, with R patches, each having m_{l-1} channels. Each attention head of dimension $d \in \mathbb{N}$ contains three weight matrices for optimisation: $\mathbf{W}_l^{\text{Query}}$, $\mathbf{W}_l^{\text{Key}}$ and $\mathbf{W}_l^{\text{Value}}$, yielding intermediate activations $\mathbf{z}_l^{\text{Query}} = \mathbf{a}_{l-1}\mathbf{W}_l^{\text{Query}}$, $\mathbf{z}_l^{\text{Key}} = \mathbf{a}_{l-1}\mathbf{W}_l^{\text{Key}}$, and $\mathbf{z}_l^{\text{Value}} = \mathbf{a}_{l-1}\mathbf{W}_l^{\text{Value}}$ respectively. The attention outputs $\mathbf{s}_l$ are then calculated as

$$\mathbf{s}_l := \text{Softmax}\left(\frac{\mathbf{z}_l^{\text{Query}} \times \left(\mathbf{z}_l^{\text{Key}}\right)^\top}{\sqrt{d}}\right) \times \mathbf{z}_l^{\text{Value}} \tag{6}$$

Forward projection may be applied to generate target values for each of $\mathbf{z}_l^{\text{Query}}, \mathbf{z}_l^{\text{Key}}, \mathbf{z}_l^{\text{Value}}$, using distinct data projection matrices $\mathbf{Q}_l^{\text{Query}}, \mathbf{Q}_l^{\text{Key}}, \mathbf{Q}_l^{\text{Value}}$, and a shared label projection matrix $\mathbf{U}_l$, such that for each patch, query matrix target potentials are given by

$$\tilde{\mathbf{z}}_{l,r}^{\text{Query}} = g_l\left(\mathbf{a}_{l-1,r}\mathbf{Q}_l^{\text{Query}}\right) + g_l\left(\mathbf{y}\mathbf{U}_l\right). \tag{7}$$

Thus, the query weight matrix is given by the penalised regression solution [7]. Weights were fitted such that

$$\mathbf{W}_l^{\text{Query}} := \left(\sum_{r=1}^{R} \mathbf{A}_{l-1,r}^\top \mathbf{A}_{l-1,r} + \lambda\mathbf{I}\right)^{-1} \left(\sum_{r=1}^{R} \mathbf{A}_{l-1,r}^\top \tilde{\mathbf{Z}}_{l,r}^{\text{Query}}\right). \tag{8}$$

$\mathbf{W}_l^{\text{Key}}$ and $\mathbf{W}_l^{\text{Value}}$, are fitted in the same manner - using $\mathbf{Q}_l^{\text{Key}}$ and $\mathbf{Q}_l^{\text{Value}}$, respectively, in place of $\mathbf{Q}_l^{\text{Query}}$ in (7).

S.5 Implementation of local learning methods

Local Supervision was implemented by generating auxiliary predictions $\hat{\mathbf{y}}_1, \ldots, \hat{\mathbf{y}}_{L-1}$ from a set of fixed linear operators $\mathbf{U}_1, \ldots \mathbf{U}_{L-1}$ such that $\hat{\mathbf{y}}_l = f_L(\mathbf{a}_l\mathbf{U}_l^+)$. In each

layer, $\mathbf{W}_l$ was optimised to minimise the auxiliary loss $\mathcal{L}_{\updownarrow}(\hat{\mathbf{y}}_l, \mathbf{y})$ via gradient descent with two layers of backpropagation, such that

$$\nabla_{\mathbf{W}_l}\mathcal{L}_l = \mathbf{a}_{l-1}^{\top}\left(f_l'(\mathbf{z}_l) \odot \left(f_L'(\mathbf{a}_l\mathbf{U}_l^{+}) \odot \nabla_{\hat{\mathbf{y}}_l}\mathcal{L}_l\right)(\mathbf{U}_l^{+})^{\top}\right). \tag{9}$$

Here f_l' denotes the derivative of f_l. Local Supervision was implemented on convolutional layers by applying global-average pooling to generate a single vector representing mean neuronal activity over all convolutional windows, such that

$$\hat{\mathbf{y}}_l = f_L\left(\frac{1}{RC}\sum_{r=1}^{R}\sum_{c=1}^{C}\mathbf{a}_{l,r,c}\mathbf{U}_l^{+}\right). \tag{10}$$

The Forward-Forward algorithm [1] was implemented by generating both "positive" data-label pairs $[\mathbf{x}, \mathbf{y}]$ and "negative" data pairs $[\mathbf{x}, \check{\mathbf{y}}]$ by concatenation. Positive activations were generated such that

$$\mathbf{a}_l^{\text{pos}} = f_l(\mathbf{W}_l \ldots f_1(\mathbf{W}_1[\mathbf{x}, \mathbf{y}]) \ldots). \tag{11}$$

Negative activations were generated with spurious labels $\check{y} \neq y$ such that

$$\mathbf{a}_l^{\text{neg}} = f_l(\mathbf{W}_l \ldots f_1(\mathbf{W}_1[\mathbf{x}, \check{\mathbf{y}}]) \ldots). \tag{12}$$

Thus, local auxiliary loss functions were computed using threshold hyperparameter $\theta = 2$ and logistic sigmoid function σ, computing auxiliary loss $\mathcal{L}_l$ such that

$$\mathcal{L}_l := \sigma(\|\mathbf{a}_l^{\text{neg}}\|_2^2 - \theta) + \sigma(\theta - \|\mathbf{a}_l^{\text{pos}}\|_2^2). \tag{13}$$

In MLP implementations, concatenation was implemented with supplementary input neurons to hold the label information as a one-hot vector. In convolutional implementations, the label was concatenated in the channel dimension, such that each channel indicated a single class, with a constant value over all kernel positions. Predictive Coding [2, 3] was implemented using inverse layers $\mathbf{W}_l^{\text{back}} \in \mathbb{R}^{m_l \times m_{l-1}}$ for each weight matrix $\mathbf{W}_l$, to estimate the pre-synaptic inputs from the post-synaptic outputs, such that

$$\hat{\mathbf{a}}_{l-1} := f_l^{\text{back}}\left(\mathbf{a}_l\mathbf{W}_l^{\text{back}}\right) \tag{14}$$

The local loss is given by the reconstruction error

$$\mathcal{L}_l := \|\mathbf{a}_{l-1} - \hat{\mathbf{a}}_{l-1}\|_2^2 \tag{15}$$

Similarly, Difference Target Propagation [4] aims to reconstruct presynaptic inputs, such that

$$\hat{\mathbf{a}}_{l-1} := \mathbf{a}_{l-1} - f_l^{\text{back}}\left(\mathbf{a}_l\mathbf{W}_l^{\text{back}}\right) + f_l^{\text{back}}\left(\hat{\mathbf{a}}_l\mathbf{W}_l^{\text{back}}\right) \tag{16}$$

In our implementations, we set $f_l^{\text{back}} = \text{ReLU}$ in both Predictive Coding and Difference Target Propagation models.

S.6 Analysis of label projection approaches

We now consider the necessity of pre-synaptic input projections, which distinguish Forward Projection from alternative feedback-free approaches such as simple label projection and noisy label projection. The objective is to generate a weight matrix $\mathbf{W}_l \in \mathbb{R}^{m_{l-1} \times m_l}$, given pre-synaptic inputs, $\mathbf{A}_{l-1} \in \mathbb{R}^{N \times m_{l-1}}$, and label matrix $\mathbf{Y} \in \mathbb{R}^{N \times m_L}$. As described previously, given some target potentials $\tilde{\mathbf{Z}}$, weights will be fitted by equation (2), such that

$$\mathbf{W}_l = (\mathbf{A}_{l-1}^\top \mathbf{A}_{l-1} + \lambda \mathbf{I})^{-1} \mathbf{A}_{l-1}^\top \tilde{\mathbf{Z}}. \tag{17}$$

We let $\text{rank}(\tilde{\mathbf{Z}})$ denote the column rank of $\tilde{\mathbf{Z}}$ – the maximum number of linearly independent column vectors in its column space. Since $\mathbf{W}_l$ is a linear function of $\tilde{\mathbf{Z}}$, we have $\text{rank}(\mathbf{W}_l) \leq \text{rank}(\tilde{\mathbf{Z}})$.

We first consider target generation by simple label projection, such that $\tilde{\mathbf{Z}}_l := \mathbf{Y}\mathbf{U}_l$. Here we have $\text{rank}(\tilde{\mathbf{Z}}) \leq \text{rank}(\mathbf{Y}) \leq m_L$. Where $m_l \gg m_L$, this implies severe degeneracy of $\mathbf{W}_l$, predisposing to correlated or redundant neuronal activities in $\mathbf{A}_l$. We next consider target generation by noisy label projection, using random Gaussian noise matrix $\mathbf{E} \in \mathbb{R}^{N \times m_l}$ to perturb targets such that $\tilde{\mathbf{Z}}_l := \mathbf{Y}\mathbf{U}_l + \mathbf{E}$. In this case, we have

$$\text{rank}(\tilde{\mathbf{Z}}_l) \leq \min\left(m_l, (\text{rank}(\mathbf{Y}) + \text{rank}(\mathbf{E}))\right) = m_l, \tag{18}$$

However, as $\mathbf{E}$ is random and independent of $\mathbf{Y}$, this perturbation strategy may adversely affect label modelling of $\mathbf{Y}$. By adding projections of label and pre-synaptic inputs, such that $\tilde{\mathbf{Z}}_l := \mathbf{A}_{l-1}\mathbf{Q}_l + \mathbf{Y}\mathbf{U}_l$, we improve the upper bound to

$$\text{rank}(\tilde{\mathbf{Z}}_l) \leq \min\left(m_l, (\text{rank}(\mathbf{A}_{l-1}) + \text{rank}(\mathbf{Y}))\right) \leq \min\left(m_l, (m_{l-1} + m_L)\right), \tag{19}$$

without fitting $\mathbf{W}_l$ to noise. Accordingly, the targets generated by our proposed method $\tilde{\mathbf{Z}}_l := g_l(\mathbf{a}_{l-1}\mathbf{Q}_l) + g_l(\mathbf{Y}\mathbf{U}_l)$ may also have full rank.

S.7 Computing the Forward Projection estimator

Neural network models often manage storage demands by utilizing data mini-batches, allowing them to train on arbitrarily large datasets without a corresponding increase in memory requirements. Although the Forward Projection estimator described in equation (2) includes large matrix terms $\mathbf{A}_{l-1} \in \mathbb{R}^{N \times m_{l-1}}$ and $\tilde{\mathbf{Z}}_l \in \mathbb{R}^{N \times m_l}$, these do not need to be loaded into memory. Assume $\mathbf{A}_{l-1} \in \mathbb{R}^{N \times m_{l-1}}$ and $\mathbf{Y} \in \mathbb{R}^{N \times m_L}$ are available as a set of row-vectors $\{\mathbf{a}_{i,l-1}\}_{i=1}^N \subset \mathbb{R}^{1 \times m_{l-1}}$ and $\{\mathbf{y}_i\}_{i=1}^N \subset \mathbb{R}^{1 \times m_L}$. The Gramian matrix is the sum of the row outer products, such that

$$\mathbf{A}_{l-1}^\top \mathbf{A}_{l-1} = \sum_{i=1}^N \mathbf{a}_{i,l-1}^\top \mathbf{a}_{i,l-1}. \tag{20}$$

Therefore, the $\mathbf{A}_{l-1}^{\top}\mathbf{A}_{l-1} \in \mathbb{R}^{m_{l-1}\times m_{l-1}}$ term may be accumulated sequentially over instances $\{\mathbf{a}_{1,l-1},\ldots,\mathbf{a}_{N,l-1}\}$, with $\mathcal{O}(m_{l-1}^2)$ memory and $\mathcal{O}(Nm_{l-1}^2)$ compute, such that

$$(\mathbf{A}_{l-1}^{\top}\mathbf{A}_{l-1})^{(i)} = (\mathbf{A}_{l-1}^{\top}\mathbf{A}_{l-1})^{(i-1)} + \mathbf{a}_{i,l-1}^{\top}\mathbf{a}_{i,l-1}, \tag{21}$$

where $(\mathbf{A}_{l-1}^{\top}\mathbf{A}_{l-1})^{(0)}$ is the zero matrix of dimension $m_{l-1}\times m_{l-1}$. We may generate the corresponding target potentials $\{\tilde{\mathbf{z}}_{i,l}\}_{i=1}^{N} \subset \mathbb{R}^{1\times m_l}$, using predetermined matrices $\mathbf{Q}_l \in \mathbb{R}^{m_{l-1}\times m_l}$ and $\mathbf{U}_l \in \mathbb{R}^{m_L\times m_l}$according to (1). Thus, $\mathbf{A}_{l-1}^{\top}\tilde{\mathbf{Z}}_l \in \mathbb{R}^{m_{l-1}\times m_l}$ may also be accumulated sequentially such that

$$\mathbf{A}_{l-1}^{\top}\tilde{\mathbf{Z}}_l = \sum_{i=1}^{N} \mathbf{a}_{i,l-1}^{\top}\tilde{\mathbf{z}}_{i,l}, \tag{22}$$

requiring $\mathcal{O}(m_{l-1}m_l)$ memory and $\mathcal{O}(Nm_{l-1}m_l)$ compute. Addition of the $\lambda\mathbf{I}$ regularisation term has negligible $\mathcal{O}(m_{l-1})$ overhead. Following sequential computation of equations (21) and (22), equation (2) may be computed with one order $\mathcal{O}(m_{l-1}^3)$ matrix inversion and one order $\mathcal{O}(m_{l-1}^2 m_l)$ matrix multiplication, yielding:

$$\mathbf{W}_l = (\mathbf{A}_{l-1}^{\top}\mathbf{A}_{l-1} + \lambda\mathbf{I})^{-1}(\mathbf{A}_{l-1}^{\top}\tilde{\mathbf{Z}}_l). \tag{23}$$

S.8 Information Encoding and Interpretability

We now consider the information encoding properties of Forward Projection training. Assuming g_l^{-1} exists everywhere, equation (1) relates labels $\mathbf{y} \in \mathbb{R}^{1\times m_L}$ to target potentials $\tilde{\mathbf{z}}_l \in \mathbb{R}^{1\times m_l}$, given presynaptic inputs $\mathbf{a}_{l-1} \in \mathbb{R}^{1\times m_{l-1}}$ via projection matrices $\mathbf{Q}_l \in \mathbb{R}^{m_{l-1}\times m_l}$ and $\mathbf{U}_l \in \mathbb{R}^{m_L\times m_l}$, such that

$$\mathbf{y}\mathbf{U}_l = g_l^{-1}\left(\tilde{\mathbf{z}}_l - g_l(\mathbf{a}_{l-1}\mathbf{Q}_l)\right). \tag{24}$$

Letting $\mathbf{U}_l^{+}$ denote the Moore-Penrose inverse, and assuming that $\mathbf{U}_l\mathbf{U}_l^{+} = \mathbf{I}$, we may reconstruct $\mathbf{y}$, such that

$$\mathbf{y} = g_l^{-1}\left(\tilde{\mathbf{z}}_l - g_l(\mathbf{a}_{l-1}\mathbf{Q}_l)\right)\mathbf{U}_l^{+}. \tag{25}$$

Assuming realised neuron pre-activation potentials $\mathbf{z}_l$ are a good approximation of the target potentials, i.e., $\mathbf{z}_l \approx \tilde{\mathbf{z}}_l$, we may estimate the label from the hidden layer, such that

$$\hat{\mathbf{y}}_l := g_l^{-1}\left(\mathbf{z}_l - g_l(\mathbf{a}_{l-1}\mathbf{Q}_l)\right)\mathbf{U}_l^{+}. \tag{26}$$

Thus, neural potentials are interpretable layer-wise as label predictions. Assuming $\mathbf{Q}_l\mathbf{Q}_l^{+} \approx \mathbf{I}$, pre-synaptic inputs may also be estimated approximately such that

$$\hat{\mathbf{a}}_{l-1} := g_l^{-1}\left(\mathbf{z}_l - g_l(\mathbf{y}\mathbf{U}_l)\right)\mathbf{Q}_l^{+}. \tag{27}$$

Thus, pre-synaptic inputs are encoded in a lossy manner in each layer's neural pre-activation potentials. In our experiments, we employed the sign function for target

generation, defined as:

$$g_l := \text{sign}(x) = \begin{cases} -1 & x < 0 \\ 0 & x = 0 \\ 1 & x > 0 \end{cases} \tag{28}$$

The element-wise function $g_l := \text{sign}(x)$ was selected due to computational simplicity and predictable distribution. The tanh function was employed as a heuristic surrogate for g_l^{-1}, yielding satisfactory results.

S.9 Stability Experiments

Technical analysis and stability experiments were performed on MLP models training on the FMNIST data. The stability of layer explanations, on models fitted to different data folds, with different random projection matrices, using a 4×1000 neuron feed-forward architecture was evaluated. Test performance of layer explanations improved progressively from the first layer to the final layer with low variability throughout (Supplementary Figure S2-A). Model stability with respect to choices of the FP penalty parameter λ (Equation 2) in hidden layers was evaluated in a 3×1000 neuron MLP. Test accuracies were stable from $\lambda = 1.25$ to $\lambda = 80$ with the highest observed performance at $\lambda = 80$ (Supplementary Figure S2-B). The mean variance of pixels in the FMNIST training dataset is $\sigma_{\text{input}} = 0.087$. Although additive gaussian noise caused progressive deterioration in model performance from $\sigma_{\text{noise}} = 0.1$ to $\sigma_{\text{noise}} = 1$, test accuracy remained above 0.819 for all $\sigma_{\text{noise}} < 0.4$, which corresponds to a signal-to-noise ratio of approximately $1 : 4.6$ (Supplementary Figure S2-C). The impact of the randomised projections on model performance was assessed in 3-layer MLP models with 2×1000 hidden neurons in the first two layers and $m_3 \in \{250, 500, 1000\}$ in the penultimate layer, with stable test performance observed throughout (Supplementary Figure S2-D).

S.10 Optimisation Theory

We now compare the ordinary least squares linear model to a two-layer network trained by Forward Projection and a Random Features model. We aim to demonstrate analytically that the expected error of the Forward Projection model improves upon the others.

Consider a dataset $\{\mathbf{x}_i, \mathbf{y}_i\}_{i=1}^N$, where $\mathbf{x}_i \sim \mathcal{N}(\mathbf{0}, \mathbf{I}_{m_l})$ and target $\mathbf{y}_i \in \mathbb{R}^{m_L}$. Let $\mathbf{X} \in \mathbb{R}^{N \times m_l}$ denote the design matrix with rows $\mathbf{x}_i^\top$ and $\mathbf{Y} \in \mathbb{R}^{N \times m_L}$ denote the target matrix with rows $\mathbf{y}_i^\top$. Let $\text{col}(\mathbf{X})$ denote the column space of matrix $\mathbf{X}$, the span of its column vectors. We assume $N > m_l \gg m_L$ and that $\mathbf{X}$ has full column rank. We first consider the expected error of a single-layer, linear model, with weights $\hat{\mathbf{W}}_{\text{linear}} \in \mathbb{R}^{m_l \times m_L}$. We estimate $\hat{\mathbf{W}}_{\text{linear}}$, by ordinary least squares [8], such that

$$\hat{\mathbf{W}}_{\text{linear}} := (\mathbf{X}^\top \mathbf{X})^{-1} \mathbf{X}^\top \mathbf{Y}. \tag{29}$$

We denote the projection matrix of $\mathbf{X}$ as

$$\mathbf{P}_{\mathbf{X}} = \mathbf{X}(\mathbf{X}^{\top}\mathbf{X})^{-1}\mathbf{X}^{\top}. \tag{30}$$

The spectrum of the idempotent projection matrix $\mathbf{P}_{\mathbf{X}}$ is composed of a unit eigenvalue with multiplicity m_l and a zero eigenvalue with multiplicity $N - m_l$ [9]. Letting $\hat{\mathbf{Y}}_{\text{linear}} = \mathbf{P}_{\mathbf{X}}\mathbf{Y}$, the model error is given by

$$E_{\text{linear}} = \mathbb{E}\left[\|\mathbf{Y} - \hat{\mathbf{Y}}_{\text{linear}}\|_F^2\right] = \mathbb{E}\left[\|(\mathbf{I} - \mathbf{P}_{\mathbf{X}})\mathbf{Y}\|_F^2\right], \tag{31}$$

Letting $\mathbf{Y}_{\perp} = (\mathbf{I} - \mathbf{P}_{\mathbf{X}})\mathbf{Y}$, we decompose [10]

$$\mathbf{Y} = \mathbf{P}_{\mathbf{X}}\mathbf{Y} + (\mathbf{I} - \mathbf{P}_{\mathbf{X}})\mathbf{Y} = \hat{\mathbf{Y}}_{\text{linear}} + \mathbf{Y}_{\perp}, \tag{32}$$

where $\text{col}(\hat{\mathbf{Y}}_{\text{linear}}) \subset \text{col}(\mathbf{X})$, and $\mathbf{Y}_{\perp}$ contains residuals. Let $\text{col}(\mathbf{X})^{\perp}$ denote the $(N - m_l)$-dimensional space orthogonal to $\text{col}(\mathbf{X})$. We note that as $(\mathbf{I} - \mathbf{P}_{\mathbf{X}})$ projects onto $\text{col}(\mathbf{X})^{\perp}$, $\mathbf{Y}_{\perp}$ lies in $\text{col}(\mathbf{X})^{\perp}$. By symmetric idempotency of $(\mathbf{I} - \mathbf{P}_{\mathbf{X}})$ and cyclic trace invariance [10], we have

$$\begin{aligned} E_{\text{Linear}} &= \mathbb{E}\left[\|\mathbf{Y}_{\perp}\|_F^2\right] = \mathbb{E}\left[\text{Tr}\left((\mathbf{I} - \mathbf{P}_{\mathbf{X}})\,\mathbf{Y}\mathbf{Y}^{\top}(\mathbf{I} - \mathbf{P}_{\mathbf{X}})^{\top}\right)\right] \\ &= \mathbb{E}\left[\text{Tr}\left((\mathbf{I} - \mathbf{P}_{\mathbf{X}})^{\top}(\mathbf{I} - \mathbf{P}_{\mathbf{X}})\,\mathbf{Y}\mathbf{Y}^{\top}\right)\right] = \mathbb{E}\left[\text{Tr}\left((\mathbf{I} - \mathbf{P}_{\mathbf{X}})\,\mathbf{Y}\mathbf{Y}^{\top}\right)\right]. \end{aligned} \tag{33}$$

We now consider a two-layer neural network model trained by forward projection for fitting the same data. The model has m_l hidden ReLU-activated neurons, such that

$$\hat{\mathbf{Y}}_{\text{FP}} := \text{ReLU}(\mathbf{X}\hat{\mathbf{W}}_1)\hat{\mathbf{W}}_2, \tag{34}$$

The task is to identify weight matrices $\hat{\mathbf{W}}_1 \in \mathbb{R}^{m_l \times m_l}, \hat{\mathbf{W}}_2 \in \mathbb{R}^{m_l \times m_L}$ to minimise the error $\|\mathbf{Y} - \hat{\mathbf{Y}}_{\text{FP}}\|_F^2$. Let $\mathbf{U} \in \mathbb{R}^{m_L \times m_l}$ and $\mathbf{Q} \in \mathbb{R}^{m_l \times m_l}$ be matrices with elements sampled from $\mathcal{N}(0, 1)$. We now fit $\hat{\mathbf{W}}_1$ to estimate $\mathbf{XQ} + \mathbf{YU}$ from $\mathbf{X}$, such that

$$\hat{\mathbf{W}}_1 := (\mathbf{X}^{\top}\mathbf{X})^{-1}\mathbf{X}^{\top}(\mathbf{XQ} + \mathbf{YU}). \tag{35}$$

Thus, the pre-activation potential at the first layer is given by

$$\mathbf{X}\hat{\mathbf{W}}_1 = \mathbf{XQ} + \mathbf{P}_{\mathbf{X}}\mathbf{YU}. \tag{36}$$

As $m_l \gg m_L$, we assume $\mathbf{U}\mathbf{U}^{+} = \mathbf{I}_{m_L}$. Independence of $\mathbf{Q}$ and $\mathbf{U}$, with elements sampled from $\mathcal{N}(0, 1)$ gives $\mathbb{E}_{\mathbf{Q},\mathbf{U}}\left[\mathbf{Q}\mathbf{U}^{+}\right] = 0$. Therefore,

$$\mathbb{E}_{\mathbf{Q},\mathbf{U}}\left[\mathbf{X}\hat{\mathbf{W}}_1\mathbf{U}^{+}\right] = \mathbb{E}_{\mathbf{Q},\mathbf{U}}\left[\mathbf{X}\mathbf{Q}\mathbf{U}^{+}\right] + \mathbb{E}_{\mathbf{U}}\left[\mathbf{P}_{\mathbf{X}}\mathbf{Y}\mathbf{U}\mathbf{U}^{+}\right] = \mathbf{P}_{\mathbf{X}}\mathbf{Y}. \tag{37}$$

Therefore, we note that in the $m_l \gg m_L$ setting, $\mathbf{P}_{\mathbf{X}}\mathbf{Y}$ is encoded by random projection over $\mathbf{X}\hat{\mathbf{W}}_1$.

Let $\mathbf{A} = \mathrm{ReLU}(\mathbf{X}\hat{\mathbf{W}}_1)$ denote the hidden activations. We note that $\mathrm{col}(\mathbf{A}) \neq \mathrm{col}(\mathbf{X})$, as the ReLU operation introduces non-linearities into $\mathbf{X}\hat{\mathbf{W}}_1$. Since $(\mathbf{Q}, \mathbf{U})$ is sampled from a symmetric distribution around zero, the realisations $(\mathbf{Q}, \mathbf{U})$ and $(-\mathbf{Q}, -\mathbf{U})$ have equal probability. Using the identity

$$\mathbf{X}\hat{\mathbf{W}}_1 = \mathrm{ReLU}(\mathbf{X}\hat{\mathbf{W}}_1) - \mathrm{ReLU}(-\mathbf{X}\hat{\mathbf{W}}_1), \tag{38}$$

we have

$$\mathbb{E}_{\mathbf{Q},\mathbf{U}}\left[\mathrm{ReLU}(\mathbf{X}\hat{\mathbf{W}}_1)\mathbf{U}^+\right] = -\mathbb{E}_{\mathbf{Q},\mathbf{U}}\left[\mathrm{ReLU}(-\mathbf{X}\hat{\mathbf{W}}_1)\mathbf{U}^+\right]. \tag{39}$$

Therefore

$$\mathbb{E}\left[\mathbf{A}\mathbf{U}^+\right] = \frac{1}{2}\mathbb{E}\left[\mathbf{X}\hat{\mathbf{W}}_1\mathbf{U}^+\right] = \frac{1}{2}\mathbf{P}_\mathbf{X}\mathbf{Y}. \tag{40}$$

In the $m_l \gg m_L$ setting, we assume convergence to the mean, and approximate $\mathbf{A}\mathbf{U}^+ \approx \frac{1}{2}\mathbf{P}_\mathbf{X}\mathbf{Y}$. Accordingly, we assume that $\mathbf{P}_\mathbf{X}\mathbf{Y}$ lies within $\mathrm{col}(\mathbf{A})$ – i.e., $\mathrm{col}(\hat{\mathbf{Y}}_{\text{linear}}) \subset \mathrm{col}(\mathbf{A})$. We also assume $\mathbf{A}$ has full column rank. We now fit $\hat{\mathbf{W}}_2$ to estimate $\mathbf{Y}$ from $\mathbf{A}$, such that

$$\hat{\mathbf{W}}_2 := (\mathbf{A}^\top\mathbf{A})^{-1}\mathbf{A}^\top\mathbf{Y}. \tag{41}$$

Letting $\hat{\mathbf{Y}}_{\text{FP}} = \mathbf{P}_\mathbf{A}\mathbf{Y}$, the Forward Projection error is given by

$$E_{\text{FP}} = \mathbb{E}\left[\|\mathbf{Y} - \hat{\mathbf{Y}}_{\text{FP}}\|_F^2\right] = \mathbb{E}\left[\|(\mathbf{I} - \mathbf{P}_\mathbf{A})\mathbf{Y}\|_F^2\right]. \tag{42}$$

From (32), we have

$$(\mathbf{I} - \mathbf{P}_\mathbf{A})\mathbf{Y} = (\mathbf{I} - \mathbf{P}_\mathbf{A})\hat{\mathbf{Y}}_{\text{linear}} + (\mathbf{I} - \mathbf{P}_\mathbf{A})\mathbf{Y}_\perp. \tag{43}$$

From our assumption that $\mathrm{col}(\hat{\mathbf{Y}}_{\text{linear}}) \subset \mathrm{col}(\mathbf{A})$, we have

$$(\mathbf{I} - \mathbf{P}_\mathbf{A})\hat{\mathbf{Y}}_{\text{linear}} = 0. \tag{44}$$

Substituting (42) and (43), we have

$$E_{\text{FP}} = \mathbb{E}\left[\|(\mathbf{I} - \mathbf{P}_\mathbf{A})\mathbf{Y}_\perp\|_F^2\right]. \tag{45}$$

As $\mathbf{P}_\mathbf{A}\mathbf{Y}_\perp$ is orthogonal to $(\mathbf{I} - \mathbf{P}_\mathbf{A})\mathbf{Y}_\perp$, we may apply the Pythagorean theorem [11], such that

$$\|\mathbf{Y}_\perp\|_F^2 = \|\mathbf{P}_\mathbf{A}\mathbf{Y}_\perp\|_F^2 + \|(\mathbf{I} - \mathbf{P}_\mathbf{A})\mathbf{Y}_\perp\|_F^2. \tag{46}$$

Substituting (33) and (42), we have

$$E_{\text{linear}} = \mathbb{E}\left[\|\mathbf{P}_\mathbf{A}\mathbf{Y}_\perp\|_F^2\right] + E_{\text{FP}}. \tag{47}$$

Therefore, under the inclusion assumption that $\mathrm{col}(\hat{\mathbf{Y}}_{\text{linear}}) \subset \mathrm{col}(\mathbf{A})$, the improvement of the Forward Projection model over the linear model is given by

$$\boxed{E_{\text{linear}} - E_{\text{FP}} = \mathbb{E}\left[\|\mathbf{P}_\mathbf{A}\mathbf{Y}_\perp\|_F^2\right] \geq 0.} \tag{48}$$

For comparison, we also consider the performance of a two-layer neural network model trained with random weights in the first layer (i.e., Random Features), such that $\mathbf{W}_{\text{random}} \in \mathbb{R}^{m_l \times m_l}$ has elements sampled from $\mathcal{N}(0,1)$. The hidden activities are given by

$$\mathbf{A}_{\text{random}} = \text{ReLU}\left(\mathbf{X}\mathbf{W}_{\text{random}}\right). \tag{49}$$

Since $\mathbf{W}_{\text{random}}$ is independent of $\mathbf{Y}$, the above hidden representation lacks alignment with the task-relevant structure that Forward Projection encodes due to the $\mathbf{YU}$ term. Hence, we cannot assume $\text{col}(\hat{\mathbf{Y}}_{\text{linear}}) \subset \text{col}(\mathbf{A}_{\text{random}})$. Lastly, due to the random selection of $\mathbf{W}_{\text{random}}$, we have

$$\mathbb{P}\left[(\mathbf{A}_{\text{random}})_{ij} = 0\right] = \mathbb{P}\left[(\mathbf{X}\mathbf{W}_{\text{random}})_{ij} \leq 0\right] = \frac{1}{2} \tag{50}$$

Therefore, $\mathbf{A}_{\text{random}}$ represents a random, nonlinear transformation of $\mathbf{X}$, with equal dimension, and reduced representational capacity due to element-wise sparsity. Only the output layer $\mathbf{W}_2$ may be trained, such that:

$$\hat{\mathbf{W}}_2 := (\mathbf{A}_{\text{random}}^\top \mathbf{A}_{\text{random}})^{-1} \mathbf{A}_{\text{random}}^\top \mathbf{Y}. \tag{51}$$

Unlike $\mathbf{A}$, which is correlated with the label, there is no reason to expect, over the distribution of $\mathbf{W}_{\text{random}}$, that $\mathbf{A}_{\text{random}}$ is a more informative design matrix than $\mathbf{X}$. Therefore, the error of this Random Features network is not expected to improve upon E_{linear}. We note that in the special case examined here, the hidden dimension is equal to the input dimension, a framework which contrasts with the typical usage of Random Features [5], where high-dimensional random projection is employed to generate a large, representative feature set. Unlike Forward Projection, which provides the improvement in (48), Random Features hidden layer activations do not provide such an expectation in a hidden layer of equal dimension to the input. Hence, this comparison reveals that ReLU nonlinearity alone is insufficient for expected improvement – the label-informed initialization is also essential.

This analysis compares a regression model with $m_l \cdot m_L$ parameters in a single layer trained by ordinary least squares regression to a two-layer neural network with $m_l(m_l + m_L)$ parameters, trained by Forward Projection; and a two-layer neural network with m_l random features and $m_l m_L$ trainable parameters. Although the Forward Projection model has the greatest number of free parameters, it is noted that these are not jointly optimised, but instead may be separated into m_l linear regressions in $\mathbf{W}_1$ and, subsequently, m_L linear regressions in $\mathbf{W}_2$.

Differences in the modelling of Forward Projection, Random Features and linear regression each present distinct profiles of advantages and limitations. Despite these architectural differences, this analysis establishes that Forward Projection offers a principled, backpropagation-free training method with expected improvement over linear regression – an expectation absent from Random Features of equal dimension.

S.11 Iterative learning with Forward Projection

An alternative to deriving the closed-form Forward Projection solution for regression over the entire dataset is to approximate the optimization procedure through iterative updates on mini-batches, while preserving local learning dynamics and target generation as formalized in Equation 1. We partition the dataset $\mathcal{D}$ into T mini-batches $\mathcal{B}_t = \{(\mathbf{x}_j, \mathbf{y}_j)\}_{j=1}^{B}$, where B is the batch size. For each mini-batch, compute $\mathbf{A}_{l-1}^{(t)} \in \mathbb{R}^{B\times m_{l-1}}$ and $\tilde{\mathbf{Z}}_l^{(t)} \in \mathbb{R}^{B\times m_l}$ using the target generation rule

$$\tilde{\mathbf{z}}_l = g_l(\mathbf{a}_{l-1}\mathbf{Q}_l) + g_l(\mathbf{y}\mathbf{U}_l). \tag{52}$$

For layer l, define the local loss:

$$\mathcal{L}_l^{(t)} = \frac{1}{B}\sum_{j=1}^{B} \|\mathbf{z}_{j,l} - \tilde{\mathbf{z}}_{j,l}\|^2, \tag{53}$$

where $\mathbf{z}_{j,l} = \mathbf{a}_{j,l-1}\mathbf{W}_l$. The gradient of $\mathcal{L}_l^{(t)}$ with respect to $\mathbf{W}_l$ is [7]:

$$\nabla_{\mathbf{W}_l}\mathcal{L}_l^{(t)} = \frac{2}{B}(\mathbf{A}_{l-1}^{(t)})^\top(\mathbf{A}_{l-1}^{(t)}\mathbf{W}_l - \tilde{\mathbf{Z}}_l^{(t)}). \tag{54}$$

Therefore, weights may be updated according to

$$\mathbf{W}_l \leftarrow \mathbf{W}_l - \eta\nabla_{\mathbf{W}_l}\mathcal{L}_l^{(t)}, \tag{55}$$

where η is the learning rate. Optionally, regularisation may be included via the ridge penalty [7]:

$$\nabla_{\mathbf{W}_l}\mathcal{L}_l^{(t)} = \frac{2}{B}\left(\mathbf{A}_{l-1}^{(t)}\right)^\top \left(\mathbf{A}_{l-1}^{(t)}\mathbf{W}_l - \tilde{\mathbf{Z}}_l^{(t)}\right) + \frac{2}{B}\lambda\mathbf{W}_l. \tag{56}$$

In experiments on FMNIST classification by MLPs with 3×1000 hidden neurons, sequential FP training achieved similar test performance to the closed-form FP solution (test AUC: $98.6 \pm 0.79\%$, test accuracy: $84.4 \pm 0.02\%$).

S.12 Modelling high-dimensional labels

We now analyse the dependence of the expected local error on the layer and label dimensions. From (26), we have,

$$\hat{\mathbf{Y}}_l := g_l^{-1}\left(\mathbf{Z}_l - g_l(\mathbf{A}_{l-1}\mathbf{Q}_l)\right)\mathbf{U}_l^{+}. \tag{57}$$

For simplicity, we analyse the special case that g_l is the identity function yielding,

$$\hat{\mathbf{Y}}_l := \left(\mathbf{Z}_l - \mathbf{A}_{l-1}\mathbf{Q}_l\right)\mathbf{U}_l^{+}. \tag{58}$$

Recalling that the realised potentials are estimates of pre-defined target potentials, such that $\mathbf{Z}_l = \tilde{\mathbf{Z}}_l - \mathbf{E}_l$, we have,

$$\hat{\mathbf{Y}}_l := \left(\tilde{\mathbf{Z}}_l - \mathbf{E}_l - \mathbf{A}_{l-1}\mathbf{Q}_l\right)\mathbf{U}_l^+. \tag{59}$$

Applying the identity function as g_l in equation (1), we have,

$$\tilde{\mathbf{Z}}_l = \mathbf{A}_{l-1}\mathbf{Q}_l + \mathbf{Y}\mathbf{U}_l. \tag{60}$$

Substituting, we have,

$$\hat{\mathbf{Y}}_l = \left(\mathbf{Y}\mathbf{U}_l - \mathbf{E}_l\right)\mathbf{U}_l^+. \tag{61}$$

Assuming that $\mathbf{U}$ has full row rank, and that $m_l \geq m_L$, we have $\mathbf{U}_l\mathbf{U}_l^+ = \mathbf{I}_{m_L}$ [12]. Therefore,

$$\hat{\mathbf{Y}}_l = \mathbf{Y} - \mathbf{E}_l\mathbf{U}_l^+. \tag{62}$$

As the realised potential is given by $\mathbf{Z}_l = \mathbf{P}_{\mathbf{A}_{l-1}}\tilde{\mathbf{Z}}_l$, the error is given by

$$\mathbf{E}_l = \tilde{\mathbf{Z}}_l - \mathbf{Z}_l = \left(\mathbf{I} - \mathbf{P}_{\mathbf{A}_{l-1}}\right)\tilde{\mathbf{Z}}_l = \left(\mathbf{I} - \mathbf{P}_{\mathbf{A}_{l-1}}\right)\left(\mathbf{A}_{l-1}\mathbf{Q}_l + \mathbf{Y}\mathbf{U}_l\right), \tag{63}$$

where $\mathbf{P}_{\mathbf{A}_{l-1}} = \mathbf{A}_{l-1}\left(\mathbf{A}_{l-1}^\top\mathbf{A}_{l-1}\right)^{-1}\mathbf{A}_{l-1}^\top$ is the projection matrix of the previous layer activity $\mathbf{A}_{l-1}$. As $\left(\mathbf{I} - \mathbf{P}_{\mathbf{A}_{l-1}}\right)\mathbf{A}_{l-1} = 0$, we have

$$\mathbf{E}_l = \left(\mathbf{I} - \mathbf{P}_{\mathbf{A}_{l-1}}\right)\mathbf{Y}\mathbf{U}_l. \tag{64}$$

The squared error is given by the squared Frobenius norm

$$\|\mathbf{E}_l\|_F^2 = \mathrm{Tr}\left(\mathbf{U}_l^\top\mathbf{Y}^\top\left(\mathbf{I} - \mathbf{P}_{\mathbf{A}_{l-1}}\right)\mathbf{Y}\mathbf{U}_l\right). \tag{65}$$

As $\mathbf{U}_l \in \mathbb{R}^{m_L \times m_l}$ has entries sampled from $\mathcal{N}(0,1)$, we have the expectation

$$\mathbb{E}_{\mathbf{U}_l}\left[\|\mathbf{E}_l\|_F^2\right] = m_l \cdot \|\left(\mathbf{I} - \mathbf{P}_{\mathbf{A}_{l-1}}\right)\mathbf{Y}\|_F^2. \tag{66}$$

Firstly, it is observed that $\mathbb{E}\left[\|\mathbf{E}_l\|_F^2\right]$ grows linearly with the number of columns in $\mathbf{Y}$, i.e. m_L. Secondly, it is observed that $\mathbb{E}\left[\|\mathbf{E}_l\|_F^2\right]$ grows linearly with the number of columns in m_l, which corresponds to equal expected error in each column of $\mathbf{Z}_l$. Assuming $\mathbf{U}_l\mathbf{U}_l^+ = \mathbf{I}_{m_L}$, we have

$$\mathbf{E}_l\mathbf{U}_l^+ = \left(\mathbf{I} - \mathbf{P}_{\mathbf{A}_{l-1}}\right)\mathbf{Y}\mathbf{U}_l\mathbf{U}_l^+ = \left(\mathbf{I} - \mathbf{P}_{\mathbf{A}_{l-1}}\right)\mathbf{Y} \tag{67}$$

Substituting into (62), and taking the squared frobenius norm we have the squared error of the label prediction

$$\mathbb{E}_{\mathbf{Q}_l,\mathbf{U}_l}\left[\|\mathbf{Y} - \hat{\mathbf{Y}}_l\|_F^2\right] = \mathbb{E}_{\mathbf{Q}_l,\mathbf{U}_l}\left[\|\mathbf{E}_l\mathbf{U}_l^+\|_F^2\right] = \|\left(\mathbf{I} - \mathbf{P}_{\mathbf{A}_{l-1}}\right)\mathbf{Y}\|_F^2 \tag{68}$$

Therefore $\mathbb{E}_{\mathbf{Q}_l,\mathbf{U}_l}\left[\|\mathbf{Y}-\hat{\mathbf{Y}}_l\|_F^2\right]$ increases with the component of $\mathbf{Y}$ orthogonal to $\text{col}(\mathbf{A}_{l-1})$. As $\text{rank}(\mathbf{A}_{l-1}) \leq m_{l-1}$, enlarging m_{l-1} may increase the capacity for $\text{col}(\mathbf{A}_{l-1})$ to represent $\mathbf{Y}$.

S.13 Training complexity of other methods

In this section, we describe the training complexity involved in each step of the different methods that are used in this paper for benchmarking purposes. Hereafter, N refers to the sample size, N_e to the number of training epochs, m the hidden layer dimension (it is assumed all layers have dimension m), m_L to label dimension and B to batch size.

S.13.1 Backpropagation

Backpropagation requires a forward pass through each layer (N_eNm^2), a backward pass to compute weight gradient (N_eNm^2) and the input gradient (N_eNm^2). After each batch, weights are updated ($N_eB^{-1}Nm^2$). For each layer, backpropation requires storage of weight parameters (m^2), input activations (Bm), and weight gradients (m^2).

S.13.2 Local Supervision

In local supervision, after the forward pass through the main layer (N_eNm^2 MACs), an auxiliary forward pass is conducted through the local supervision head (N_eNmm_L MACs). Subsequently, the auxiliary gradient is computed at the local supervision head (N_eNmm_L MACs), which is backpropagated through the main layer (N_eNm^2 MACs). Forward weight updates for the main layer occur at the end of each batch ($N_eB^{-1}Nm^2$ MACs). Optionally, the auxiliary layer may be trained, requiring gradient computation (N_eNmm_L MACs) and weight updates ($N_eB^{-1}Nmm_L$ MACs). Local supervision requires storage of main layer weights (m^2), auxiliary layer weights (mm_L), main layer input activations (Bm), main layer output activations (Bm), auxiliary layer output activations (Bm_L) and weight updates (m^2).

S.13.3 Forward-Forward (FF)

For a given layer, FF requires two forward passes – for "positive" and "negative" data, respectively ($2N_eNm^2$ MACs) [1]. A goodness metric is computed for both positive and negative activations, requiring $2N_eNm$ MACs. Local gradients are computed for both positive and negative passes ($2N_eNm^2$ MACs). Forward weight updates occur with each batch ($N_eB^{-1}Nm^2$ MACs). At inference time, forward passes are required for each possible class label (Nm^2m_L MACs). Forward-Forward requires storage of weight parameters (m^2), positive and negative input activations ($2Bm$), positive and negative output activations ($2Bm$) and weight updates (m^2).

S.13.4 Difference Target Propagation (DTP)

DTP replaces gradient transport with targets formed using a learned approximate inverse (decoder) and a difference correction. Training requires an encoder forward

pass ($N_e N m^2$ MACs), and two decoder passes ($2N_e N m^2$ MACs) to perform the difference correction operation between targets and observed activations [4]. Decoder training employs a denoising style loss, forwarding through both encoder ($N_e N m^2$ MACs) and decoder ($N_e N m^2$ MACs), before computing the decoder weight gradient ($N_e N m^2$ MACs). Finally, the encoder weight gradient is computed with respect to the local target loss ($N_e N m^2$ MACs). Weight updates for both encoder and decoder occur after each batch ($2N_e B^{-1} N m^2$ MACs). At inference time, only the encoder forward pass is required. Assuming batch size $B = 1$, training requires a forward pass ($N_e N m^2$ MACs) and $8N_e N m^2$ further MACs per layer. DTP requires storage of encoder and decoder weight parameters ($2m^2$), input activations (Bm), output activations (Bm), targets (Bm) and weight updates (m^2), which can be performed sequentially for encoder and decoder.

S.13.5 Predictive Coding

PC performs k iterations of activity inference that alternate forward prediction ($kN_e N m^2$ MACs) and feedback error projection through a decoder network ($kN_e N m^2$ MACs), followed by a local Hebbian weight gradient computation ($N_e N m^2$ MACs) [3]. Forward weight updates occur at the end of each batch ($N_e B^{-1} N m^2$ MACs). Letting $k = 1$, $B = 1$, training requires a forward pass ($N_e N m^2$ MACs) and $3N_e N m^2$ further MACs per layer. Predictive coding requires storage of weight parameters (m^2), input activations (Bm), output activations (Bm), output targets (Bm) and hebbian weight updates (m^2).

S.14 Modelling with Challenging Hidden Activation Functions

Neural networks commonly employ simple activation functions such as ReLU, due to low computational complexity and favourable approximation properties. Alternative hidden activation functions in sigmoid, polynomial and modulo families are desirable for modelling various physical and theoretical systems [13]. However, problems such as gradient vanishing and saturation may arise when these activation functions are trained via SGD-based methods [13, 14]. SGD-based training is not directly applicable where f_l is undifferentiable, necessitating the use of surrogate gradient methods. Many undifferentiable activation functions, such as the Heaviside step function, have attractive properties of low computational complexity and biological plausibility. Forward Projection does not require hidden activation functions to be differentiable, as target potentials $\tilde{\mathbf{Z}}_1, \dots, \tilde{\mathbf{Z}}_{l-1}$ are modelled before activation. Thus, Forward Projection presents many opportunities for modelling activation functions for which SGD-based training is intractable. Going beyond the standard activation functions, we evaluated the performance of functions which present a challenge for SGD-based training. Networks were modelled with modulo 2 activation ("mod2": $f(x) = x \mod 2$) and "square" activation $f(x) = x^2$. To control the desired range for the target potentials, an additive constant $\alpha \in \mathbb{R}$ was included in the target generation function, such that

$$\tilde{\mathbf{z}}_l = \text{sign}(\mathbf{a}_{l-1}\mathbf{Q}_l) + \text{sign}(\mathbf{y}\mathbf{U}_l) + \alpha. \tag{69}$$

Dataset	Method / Activation	Forward projection (Ours)	Random features	Local Supervision	Forward Forward	Backprop. (reference standard)
FMNIST	mod2	60.9 ± 0.4	34.4 ± 0.4	10.0 ± 0.2	9.8 ± 0.3	9.9 ± 0.2
	square	86.0 ± 0.3	67.0 ± 0.2	81.5 ± 1.4	38.6 ± 39.1	65.9 ± 6.2
Promoters	mod2	76.7 ± 0.4	49.7 ± 0.6	50.0 ± 0.0	50.2 ± 0.5	49.9 ± 0.2
	square	78.4 ± 6.2	50.0 ± 0.0	52.2 ± 3.0	54.4 ± 9.8	54.5 ± 5.7
PTBXL-MI	mod2	76.6 ± 2.5	50.0 ± 0.3	50.0 ± 0.0	48.5 ± 2.2	50.0 ± 0.0
	square	83.3 ± 1.5	50.0 ± 0.0	51.0 ± 2.0	64.6 ± 4.7	51.4 ± 1.6

Table S1: Test accuracies of local learning methods in FashionMNIST, Promoters and PTBXL-MI tasks, using modulo 2 ("mod2") ($f(x) = x \mod 2$) and square ($f(x) = x^2$) activation functions. FP: Forward Projection; LS: Local Supervision; FF: Forward-Forward; BP: Backpropagation

This hyperparameter was predefined as $\alpha = 0.5$ for both mod2 and square implementations. Forward projection yielded consistent performance across various activation functions in each dataset. In contrast, attempts at SGD-based training failed to converge in each task. Performance of Forward Projection, random features, Local Supervision, Forward-Forward and backpropagation modelling with square and mod2 activations is provided in Supplementary Table S1.

S.15 Few-shot modelling

Discrimination performance of Difference Target Propagation and Predictive Coding methods in few-shot learning tasks is plotted in Supplementary Figure S3. Both methods proved uninformative for few-shot learning CXR (Supplementary Figure S3-A), OCT (Supplementary Figure S3-B), and CIFAR2 tasks (Supplementary Figure S3-C).

Discrimination performance (AUC) of Forward Projection, random features, Local Supervision, Forward-Forward, Predictive Coding, Difference Target Propagation and and backpropagation in few-shot modelling tasks is provided in Supplementary Table S2.

S.16 Training Times

Training times and epochs for each method, in full dataset modelling. FMNIST was modelled with a 3×1000 neuron MLP network. Sequential datasets (PTBXL-MI and Promoters) were modelled by a 1D-CNN architecture of four convolutional blocks with $32 \times 2^{l-1}$ filters in the l-th block. CIFAR2 modelling used a vision transformer architecture operating on image patches of dimension 4×4, with a sequential stack of four multi-headed attention layers, each having 8 heads, embedding dimension 64, and MLP dimension 64. All experiments were run on the Google Colab service using an NVIDIA T4 graphics processing unit.

									AUC
		Method	FP (ours)	RF	LS	FF	PC	DTP	BP
Dataset	Partition	N_{train}							
CXR	Train	N=5	100.0 ± 0.0	100.0 ± 0.0	50.7 ± 16.0	52.0 ± 9.5	51.0 ± 12.9	53.9 ± 12.7	72.5 ± 9.4
		N=10	100.0 ± 0.0	100.0 ± 0.0	48.7 ± 12.7	48.8 ± 6.3	49.4 ± 9.9	53.8 ± 10.4	69.0 ± 8.1
		N=15	100.0 ± 0.0	100.0 ± 0.0	55.2 ± 13.9	51.1 ± 6.1	49.4 ± 9.5	50.4 ± 8.8	72.4 ± 10.6
		N=20	100.0 ± 0.0	100.0 ± 0.2	58.5 ± 12.8	50.0 ± 5.5	50.2 ± 9.6	52.0 ± 7.4	72.6 ± 8.0
		N=30	100.0 ± 0.0	99.9 ± 0.4	59.8 ± 15.5	50.0 ± 5.7	52.4 ± 9.0	49.4 ± 8.4	71.8 ± 9.6
		N=40	100.0 ± 0.1	99.3 ± 0.8	61.4 ± 15.8	49.7 ± 4.8	52.1 ± 8.9	52.3 ± 7.9	74.3 ± 10.2
		N=50	99.9 ± 0.2	98.4 ± 1.0	66.1 ± 14.8	49.4 ± 6.2	51.4 ± 8.2	52.2 ± 7.3	84.2 ± 9.8
	Test	N=5	75.2 ± 7.0	67.6 ± 7.5	49.7 ± 8.8	49.3 ± 5.7	50.2 ± 8.1	51.8 ± 7.6	57.6 ± 7.6
		N=10	76.6 ± 5.8	68.1 ± 6.0	47.9 ± 9.0	49.1 ± 5.7	49.3 ± 8.9	52.7 ± 8.3	59.7 ± 6.2
		N=15	77.4 ± 4.5	69.2 ± 4.8	53.9 ± 12.3	49.9 ± 6.7	49.0 ± 8.3	50.2 ± 7.6	63.1 ± 8.6
		N=20	78.9 ± 3.7	69.0 ± 4.9	58.9 ± 11.5	49.0 ± 5.8	49.6 ± 8.5	51.0 ± 7.1	66.1 ± 9.3
		N=30	80.6 ± 3.7	69.5 ± 3.7	59.2 ± 14.0	51.2 ± 6.2	51.9 ± 8.8	49.1 ± 8.9	66.6 ± 8.0
		N=40	81.7 ± 2.9	72.4 ± 3.7	61.2 ± 16.1	48.0 ± 6.4	50.3 ± 7.6	51.3 ± 8.6	70.2 ± 8.3
		N=50	82.5 ± 3.5	72.2 ± 3.1	64.8 ± 14.9	49.4 ± 7.4	50.1 ± 7.9	50.9 ± 7.2	78.9 ± 8.3
OCT	Train	N=5	100.0 ± 0.0	100.0 ± 0.0	53.9 ± 27.2	49.4 ± 12.5	52.5 ± 23.0	56.9 ± 21.5	86.8 ± 13.2
		N=10	100.0 ± 0.0	100.0 ± 0.0	58.3 ± 17.8	50.4 ± 12.2	51.4 ± 15.9	54.4 ± 13.8	83.3 ± 12.2
		N=15	100.0 ± 0.0	100.0 ± 0.0	57.1 ± 15.7	49.9 ± 11.2	49.6 ± 12.3	52.5 ± 13.8	80.5 ± 11.2
		N=20	100.0 ± 0.0	100.0 ± 0.0	53.4 ± 21.0	51.6 ± 9.8	52.9 ± 13.0	52.8 ± 11.7	88.9 ± 12.1
		N=30	100.0 ± 0.0	100.0 ± 0.0	58.0 ± 18.8	50.1 ± 11.3	50.1 ± 11.5	51.7 ± 9.7	83.7 ± 12.3
		N=40	100.0 ± 0.0	99.9 ± 0.3	61.4 ± 21.0	52.3 ± 9.2	52.2 ± 11.2	51.0 ± 12.6	88.0 ± 12.0
		N=50	100.0 ± 0.0	99.7 ± 0.7	61.7 ± 19.8	50.8 ± 13.6	51.1 ± 8.8	53.6 ± 11.0	95.2 ± 8.3
	Test	N=5	76.3 ± 11.5	63.1 ± 10.0	51.1 ± 17.6	52.5 ± 12.0	50.1 ± 15.6	47.1 ± 10.5	61.8 ± 17.4
		N=10	84.5 ± 5.8	66.5 ± 7.7	54.6 ± 16.7	51.8 ± 12.1	49.0 ± 17.1	49.2 ± 11.6	71.8 ± 15.9
		N=15	86.5 ± 7.8	68.7 ± 7.4	56.8 ± 16.3	47.8 ± 12.8	49.6 ± 13.6	52.2 ± 12.9	72.3 ± 14.4
		N=20	88.4 ± 5.9	69.5 ± 6.0	49.6 ± 19.1	52.3 ± 13.0	50.2 ± 15.5	49.9 ± 14.7	82.4 ± 14.4
		N=30	89.9 ± 4.0	70.5 ± 5.7	55.7 ± 20.9	51.8 ± 11.7	49.6 ± 15.5	48.4 ± 13.9	81.9 ± 12.1
		N=40	90.2 ± 5.0	73.3 ± 5.5	62.0 ± 25.1	50.6 ± 13.0	51.6 ± 16.5	51.1 ± 15.4	88.7 ± 9.3
		N=50	91.2 ± 4.7	73.6 ± 5.9	62.6 ± 23.8	49.7 ± 18.4	51.2 ± 12.9	51.2 ± 13.4	94.2 ± 6.6
CIFAR	Train	N=25	95.3 ± 2.7	100.0 ± 0.0	71.6 ± 23.6	52.6 ± 9.7	49.6 ± 17.5	55.9 ± 16.2	96.8 ± 4.5
		N=50	96.0 ± 2.0	100.0 ± 0.0	78.9 ± 18.3	59.2 ± 21.0	60.3 ± 16.5	56.1 ± 13.8	99.4 ± 1.9
		N=75	96.0 ± 1.6	100.0 ± 0.0	86.7 ± 5.2	67.5 ± 17.8	64.6 ± 15.1	63.4 ± 11.4	98.2 ± 2.5
		N=100	95.9 ± 1.3	100.0 ± 0.1	88.7 ± 3.8	73.7 ± 10.6	69.6 ± 11.0	60.9 ± 8.9	99.9 ± 0.3
	Test	N=25	78.6 ± 3.4	78.6 ± 3.6	65.0 ± 20.5	52.5 ± 8.8	47.7 ± 14.6	54.2 ± 14.1	82.2 ± 2.4
		N=50	83.1 ± 2.8	77.3 ± 3.1	74.2 ± 16.5	60.1 ± 22.8	58.5 ± 16.3	54.6 ± 13.4	86.3 ± 2.2
		N=75	84.4 ± 1.7	76.2 ± 3.1	81.5 ± 2.6	68.3 ± 18.8	64.4 ± 15.0	62.8 ± 10.7	87.5 ± 2.1
		N=100	86.1 ± 2.0	78.1 ± 2.6	83.5 ± 2.8	74.3 ± 9.7	68.6 ± 11.9	60.7 ± 9.4	89.5 ± 1.4

Table S2: Train and Test AUC performance in few-shot learning experiments. AUC: area under curve. CXR: chest x-ray dataset. OCT: optical coherence tomography dataset. BP: Backpropagation; DTP: Difference Target Propagation; FF: Forward Forward; FP: Forward Projection; LS: Local Supervision; PC: Predictive Coding; RF: Random features

	Method	FP	RF	LS	FF	PC	DTP	BP
Dataset	Metric							
FMNIST	Time (s)	0.3 ± 0.1	0.1 ± 0.0	46.9 ± 12.5	171.9 ± 56.6	150.2 ± 61.2	72.7 ± 26.4	22.4 ± 4.8
Promoters		6.5 ± 0.2	3.3 ± 0.6	91.6 ± 37.7	69.5 ± 12.4	258.9 ± 233.7	103.0 ± 23.6	30.9 ± 4.3
PTBXL-MI		10.6 ± 1.1	6.7 ± 0.8	56.3 ± 13.8	35.5 ± 11.9	73.3 ± 15.8	61.0 ± 24.2	18.7 ± 5.1
CIFAR2		16.3 ± 0.6	9.2 ± 0.6					43.3 ± 15.1
FMNIST	Epochs	1.0 ± 0.0	1.0 ± 0.0	10.8 ± 3.6	40.2 ± 13.8	25.4 ± 10.2	13.6 ± 5.3	11.6 ± 2.7
Promoters		1.0 ± 0.0	1.0 ± 0.0	11.4 ± 4.9	6.2 ± 1.3	18.4 ± 17.5	7.8 ± 2.0	10.0 ± 1.4
PTBXL-MI		1.0 ± 0.0	1.0 ± 0.0	17.0 ± 4.3	7.2 ± 2.3	14.6 ± 3.3	13.8 ± 5.9	13.2 ± 3.6
CIFAR		1.0 ± 0.0	1.0 ± 0.0					10.8 ± 3.9

Table S3: Training times and epochs in full dataset modelling experiments. BP: Backpropagation; DTP: Difference Target Propagation; FF: Forward Forward; FP: Forward Projection; LS: Local Supervision; PC: Predictive Coding; RF: Random features

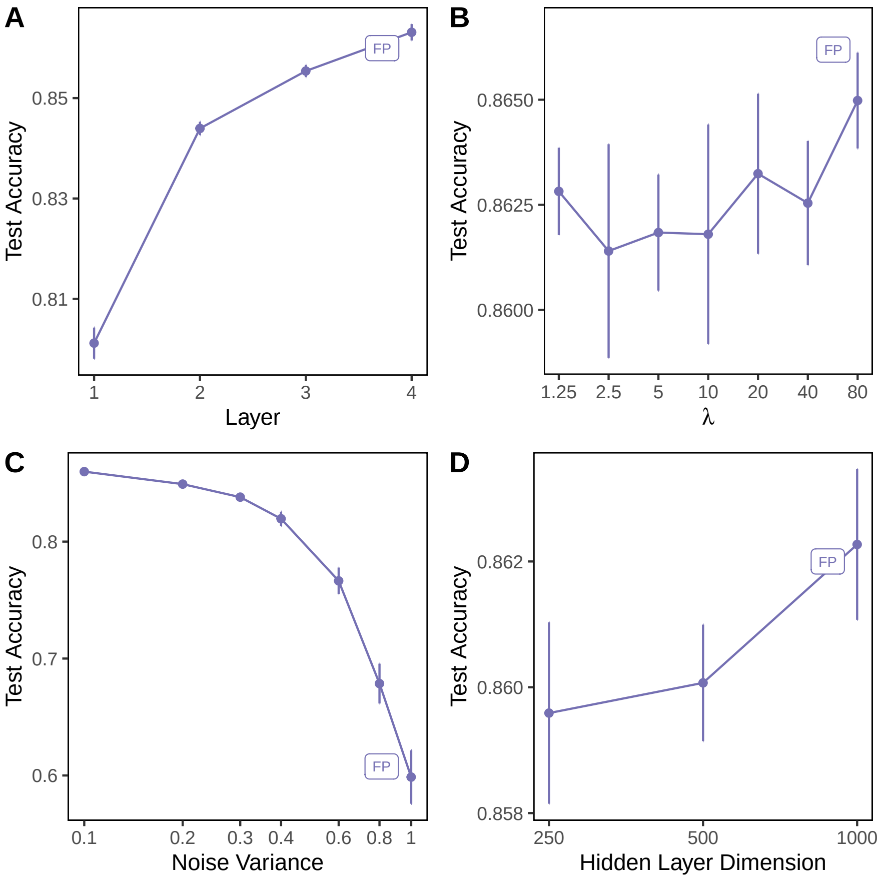

Fig. S2: Technical analysis of multilayer perceptron trained by Forward Projection. A: Test accuracy of hidden layer explanations in a 4×1000 neuron MLP trained by FP on FMNIST. B: Test accuracy of 3×1000 neuron MLP trained trained by FP on FMNIST using different λ regularisation values. C: Test accuracy of 3×1000 neuron MLP trained trained by FP on FMNIST with additive Gaussian noise in training data. D: Variability of FP training performance using different random projections in 3×1000 neuron MLP on FMNIST. Points represent mean over all replicates and error bars represent $\pm$ one standard deviation. BP: backpropagation; DTP: Difference Target Propagation; FF: Forward-Forward; FP: Forward Projection; LP: label projection; LPN: noisy label projection; LS: Local Supervision; PC: Predicitve Coding; RF: Random Features.

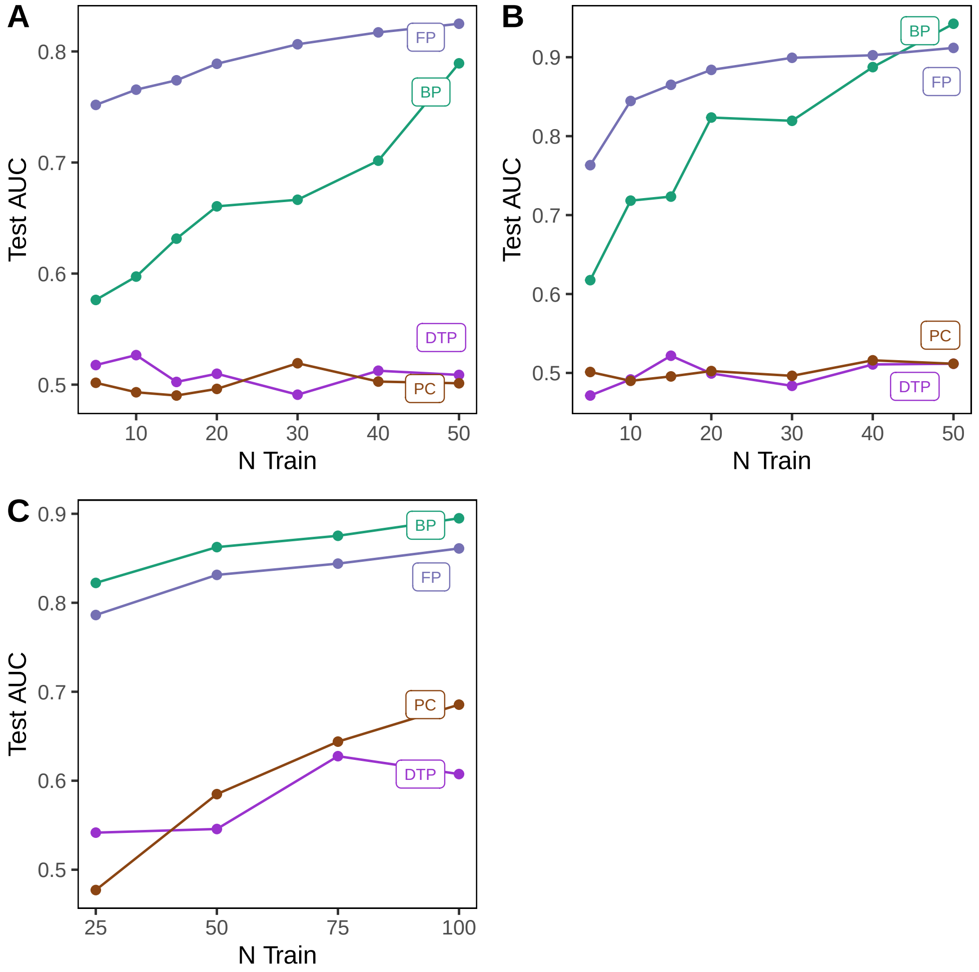

Fig. S3: Test performance of few-shot trained 2D-CNN models, showing Difference Target Propagation (DTP) and Predictive Coding (PC) methods. Mean test AUC is reported over 50 few-shot training experiments. A: Chest X-ray (CXR) task. B: Optical Coherence Tomography (OCT) task. C: CIFAR2 task in which models were required to classify the first two classes (aeroplane and automobile). Mean test AUC is reported over 50 few-shot training experiments. Models were fitted with $N \in \{5, 10, 15, 20, 30, 40, 50\}$ training samples from each class in CXR and OCT tasks and and $N \in \{25, 50, 75, 100\}$ samples per class for the CIFAR2 task.

Supplementary References

[1] Hinton, G. E. The forward-forward algorithm: Some preliminary investigations. *CoRR* **abs/2212.13345** (2022). URL https://doi.org/10.48550/arXiv.2212.13345.

[2] Friston, K. Hierarchical models in the brain. *PLoS Computational Biology* **4**, e1000211 (2008). URL http://dx.doi.org/10.1371/journal.pcbi.1000211.

[3] Millidge, B., Seth, A. K. & Buckley, C. L. Predictive coding: a theoretical and experimental review. *CoRR* **abs/2107.12979** (2021). URL https://arxiv.org/abs/2107.12979.

[4] Lee, D., Zhang, S., Fischer, A. & Bengio, Y. Appice, A. *et al.* (eds) *Difference target propagation.* (eds Appice, A. *et al.*) *Machine Learning and Knowledge Discovery in Databases - European Conference, ECML PKDD 2015, Porto, Portugal, September 7-11, 2015, Proceedings, Part I*, Vol. 9284 of *Lecture Notes in Computer Science*, 498–515 (Springer, 2015). URL https://doi.org/10.1007/978-3-319-23528-8_31.

[5] Rahimi, A. & Recht, B. Platt, J. C. *et al.* (eds) *Random features for large-scale kernel machines.* (eds Platt, J. C. *et al.*) *Proc. Advances in Neural Information Processing Systems 20*, 1177–1184 (Curran, 2007). URL https://proceedings.neurips.cc/paper/2007/hash/013a006f03dbc5392effeb8f18fda755-Abstract.html.

[6] Vaswani, A. *et al.* Guyon, I. *et al.* (eds) *Attention is all you need.* (eds Guyon, I. *et al.*) *Advances in Neural Information Processing Systems 30: Annual Conference on Neural Information Processing Systems 2017, December 4-9, 2017, Long Beach, CA, USA*, 5998–6008 (2017). URL https://proceedings.neurips.cc/paper/2017/hash/3f5ee243547dee91fbd053c1c4a845aa-Abstract.html.

[7] Golub, G. H. & Van Loan, C. F. *Matrix Computations* 4 edn. Johns Hopkins Studies in the Mathematical Sciences (Johns Hopkins University Press, Baltimore, MD, 2013).

[8] Anderson, T. W. *An introduction to multivariate statistical analysis* 3 edn. Wiley Series in Probability and Statistics (John Wiley & Sons, Nashville, TN, 2003).

[9] Meyer, C. *Matrix Analysis and Applied Linear Algebra* (SIAM, 3600 Market Street, 6th Floor Philadelphia, PA 19104-2688, 2000).

[10] Horn, R. A. & Johnson, C. R. *Matrix Analysis* (Cambridge University Press, 2012). URL http://dx.doi.org/10.1017/CBO9781139020411.

[11] Kreyszig, E. *Introductory functional analysis with applications* Wiley Classics Library (John Wiley & Sons, Nashville, TN, 1989).

[12] Penrose, R. A generalized inverse for matrices. *Mathematical Proceedings of the Cambridge Philosophical Society* **51**, 406–413 (1955). URL http://dx.doi.org/10.1017/S0305004100030401.

[13] Wang, J., Chen, L. & Ng, C. W. W. Candan, K. S., Liu, H., Akoglu, L., Dong, X. L. & Tang, J. (eds) *A new class of polynomial activation functions of deep learning for precipitation forecasting.* (eds Candan, K. S., Liu, H., Akoglu, L., Dong, X. L. & Tang, J.) *WSDM '22: The Fifteenth ACM International Conference on Web Search and Data Mining, Virtual Event / Tempe, AZ, USA, February 21 - 25, 2022*, 1025–1035 (ACM, 2022). URL https://doi.org/10.1145/3488560.3498448.

[14] Glorot, X. & Bengio, Y. Teh, Y. W. & Titterington, D. M. (eds) *Understanding the difficulty of training deep feedforward neural networks.* (eds Teh, Y. W. & Titterington, D. M.) *Proceedings of the Thirteenth International Conference on Artificial Intelligence and Statistics, AISTATS 2010, Chia Laguna Resort, Sardinia, Italy, May 13-15, 2010*, Vol. 9 of *JMLR Proceedings*, 249–256 (JMLR.org, 2010). URL http://proceedings.mlr.press/v9/glorot10a.html.
